# Association of insulin resistance and health-related quality of life with mild cognitive impairment during aging

**DOI:** 10.1080/02813432.2026.2686715

**Published:** 2026-06-23

**Authors:** Sanna Rotonen, Juha Auvinen, Aini Bloigu, Pirjo Härkönen, Jari Jokelainen, Markku Timonen, Sirkka Keinänen-Kiukaanniemi

**Affiliations:** ^a^ Research Unit of Population Health, Faculty of Medicine, University of Oulu, Finland; ^b^ Medical Research Center Oulu, Oulu University Hospital, Oulu, Finland; ^c^ Northern Finland Birth Cohorts, Arctic Biobank, Infrastructure for Population Studies, Faculty of Medicine, University of Oulu, Oulu, Finland; ^d^ Wellbeing Services County of Northern Ostrobothnia, Unit of Primary Care, Oulu, Finland; ^e^ Wellbeing Services County of Lapland

**Keywords:** Insulin resistance, cognitive performance, cognitive impairment, health-related quality of life, physical activity

## Abstract

**Background:**

Previous studies have shown an association between dementia and diabetes. This study focused on examining whether insulin resistance is a risk factor for mild cognitive impairment (MCI). We also evaluated whether common cardiovascular risk factors or depressive symptoms modify the risk of MCI along with insulin resistance and compared the health-related quality of life (HRQoL) with or without MCI.

**Methods:**

The participants were followed from 57 to 69 years of age and divided into two groups, MCI and non-MCI, according to their Consortium to Establish a Registry for Alzheimer’s Disease **(**CERAD) total score at the follow-up. The changes in Homeostatic Model Assessment of Insulin Resistance (HOMA-IR) and fasting insulin level during the follow-up were determined. Smoking, alcohol consumption, physical activity, depressive symptoms, and HRQoL were obtained by questionnaire. Body weight, height, blood pressure, and low-density lipoprotein (LDL) cholesterol were measured at baseline and follow-up. Adjustments were made for baseline cognitive performance, professional education and physical activity.

**Results:**

Greater increases in HOMA-IR (OR 2.20, 95% CI 1.05–4.62) and fasting insulin (OR 3.49, 95% CI 1.48–8.23) increased the risk of MCI. Lower cognitive performance at baseline (OR 6.33; 95% CI 3.27–12.29), lower level of education (OR 3.94, 95% CI 1.76–8.81) and lower level of physical activity (OR 2.44, 95% CI 1.06–5.65) also increased the risk of MCI at follow-up. There was a greater decline in health-related quality of life among the MCI group.

**Conclusion:**

Insulin resistance is a risk factor for MCI and MCI causes worsening of HRQoL during aging.

## Introduction

Numerous epidemiological and clinical studies have shown that both glucose metabolism disorders and disorders in cognitive performance become more common with ageing [1–3]. Prediabetes is a mild glucose metabolism disorder preceding diabetes while mild cognitive impairment (MCI) represents a transitional stage between healthy aging and dementia. People with MCI have a greater risk of developing Alzheimer′s disease or dementia compared to those who are cognitively intact [4,5]. The clinical criteria for amnestic MCI are subjective memory complaint, objective memory impairment for age, essentially preserved general cognitive function, largely intact functional activities and lack of dementia. The functional impairment is usually so mild that it is difficult to distinguish possible functional problems from those encountered during aging in general [6].

Previous studies have shown that insulin resistance plays a diverse role in brain aging and can accelerate the memory deficits possibly leading to dementia during aging [7–12]. There have been few longitudinal studies on the association between increasing insulin resistance and accelerated decline or cognitive performance in non-diabetic populations and the mechanism behind this possible association is unclear [13–17].

Previous studies concerning the life quality of patients with Alzheimer′s disease have had limited data on MCI [18]. According to a recent study by Aye et al. anxiety/depression and pain/discomfort are the most affected life quality domains, whereas mobility, self-care, and usual activity are relatively unaffected in individuals with subjective cognitive decline and MCI [19].

The aim of the present study was to evaluate the association between insulin resistance and MCI (i.e. pre-stage dementia) in a 12-year follow-up of the general population. We also evaluated whether common cardiovascular risk factors (i.e. smoking, Body mass index (BMI), blood pressure, low-density lipoprotein (LDL) cholesterol, alcohol consumption, and sedentary lifestyle) or depressive symptoms modify the risk of MCI along with insulin resistance. Our hypothesis was that insulin resistance plays a significant role in the development of MCI, which is known to be a risk factor for dementia. In addition, we wanted to investigate the health-related quality of life (HRQoL) among patients diagnosed with MCI. We also examined whether insulin resistance affects HRQoL.

## Materials and methods

### Study population and design

**Figure 1.**
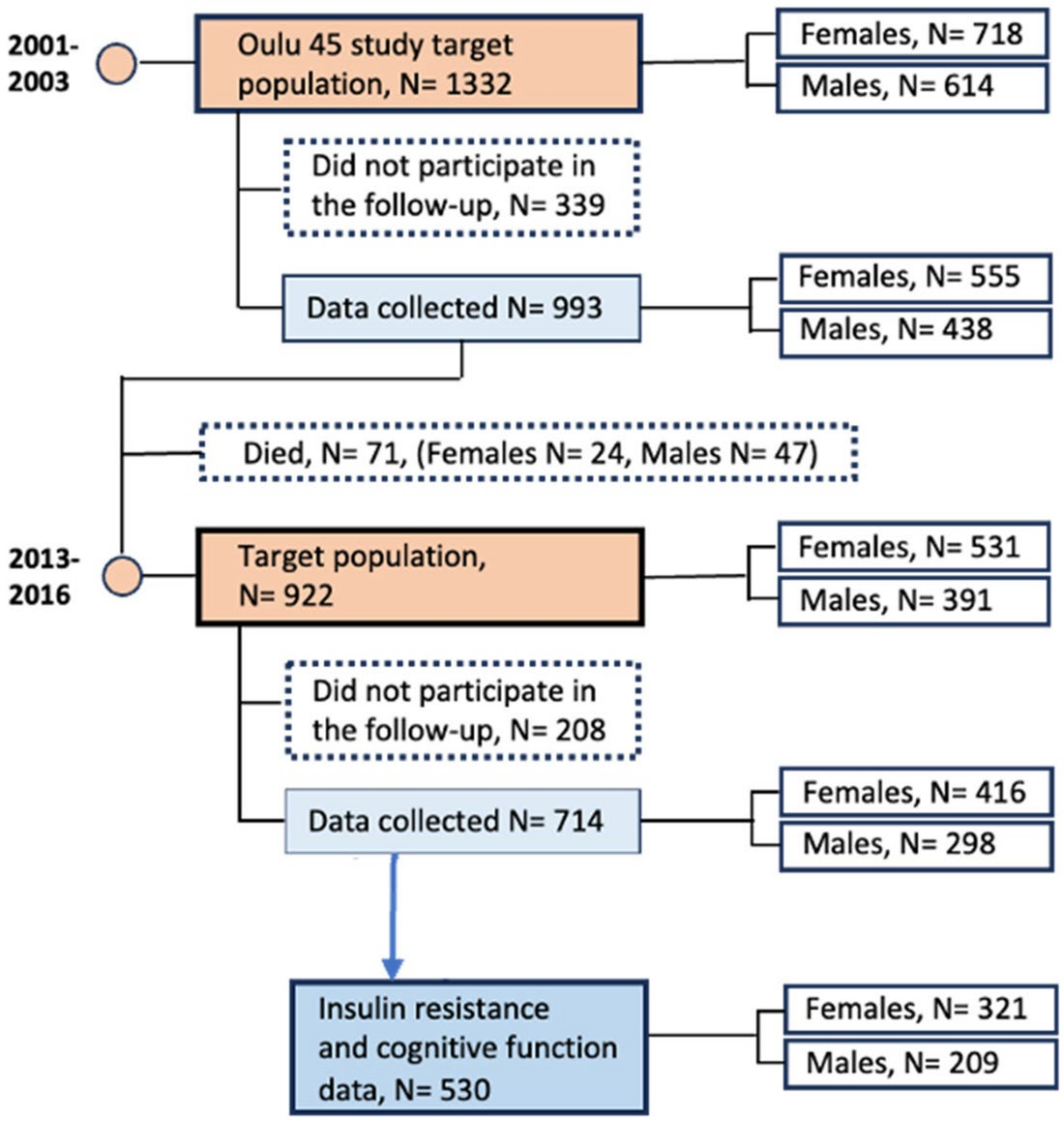
The flowchart of the study.

The population of this study was the Oulu 45 cohort, a population-based health survey and study on the aging population. Originally, all 1332 individuals born in 1945 who were living in Oulu, Finland, in 2001 (Oulu 45 cohort) were invited to participate in the baseline clinical examination from 2001–2003, and 993 (75%) of them participated. The Oulu 45 cohort was followed up from 57 (SD 0.5) years old (in 2001–2003) to 69 (SD 0.5) years old (in 2013–2015). The average follow-up was 12.1 (SD 0.5) years. In 2013–2015, 922 baseline participants were alive and invited to participate in a follow-up: 714 agreed to participate [20]. A total of 279 dropped out from the follow-up, and 161 of them had normal glucose tolerance (NGT) at baseline. Those who had been diagnosed with diabetes or were prescribed diabetes medication at baseline (*n* = 39), and those who were missing data on fasting glucose or fasting insulin (*n* = 22), Consortium to Establish a Registry for Alzheimer’s Disease (CERAD) test results (*n* = 54), or some essential variable (*n* = 69) were excluded from the analysis. The final study population consisted of 209 men and 321 women. The study flowchart is presented in Figure 1.

The characteristics of individuals who were excluded from the final analysis due to missing data (*n* = 184) differed from those of the final study population in terms of professional education: 39.1% of the excluded participants had no professional education, compared with 36.6% in the study population. Glucose status was normal in 67.9% of those excluded, whereas 80.6% of the study population had normal glucose status.

### Demographic factors and clinical measurements

The data were collected by trained study nurses through interviews, structured questionnaires on health and well-being (including physical activity, smoking habits, and alcohol consumption), and the following clinical measurements at both baseline and follow-up: cognitive tests, weight, height, waist and hip circumference, blood pressure, heart rate, arterial stiffness [21], and overnight fasted blood samples. The LDL cholesterol was measured at both baseline and follow-up. Clinical measurements were performed on two separate days. The first examination day included the basic anthropometric and clinical measurements and blood sampling after overnight fasting. The second examination day included cognitive function and performance tests in a restful room without rushing at noon or in the afternoon.

The Beck Depression Inventory (BDI) [22] was used to examine depressive symptoms among the study population. A score of 0–9 indicates that a person does not have depressive symptoms exceeding the clinical cut-off., and a score ≥ 10 indicates mild-moderate, moderate-severe, or severe depressive symptoms. Based on BDI cut-off scores, we used < 10 as a cut-off for no depressive symptoms.

All individuals in the study population had at least 4–8 years of elementary school as a basic education. No professional education was obtained after that basic education by 194 participants, 115 participants had 9–12 years of education (vocational education), and 221 had > 12 years (college, polytechnic, academy, or university degree) of basic and professional education.

Participants reported their physical activity, and the population was divided into two groups: physical activity less than 1 time per week and physical activity once a week or more often. The first group was defined as a low level of physical activity.

### Definition of mild cognitive impairment

The cognitive tests applied at baseline were the verbal fluency tests category and letter, the trail making tests parts A and B (TMA and TMB, respectively), and the word list learning. At follow-up, the study population completed the entire CERAD test battery, the TMA and TMB [23], and The Mini-Mental State Examination (MMSE) [24]. The test battery applied at baseline was less extensive than the battery applied at follow-up due to the young age and generally good health and well-being of the population at baseline. According to previous findings, the CERAD total score is a highly accurate measure for discriminating between healthy elderly subjects and subjects with MCI [25]. According to a meta-analysis by Breton et al. in 2018, the use of comprehensive cognitive tests remains desirable for diagnostic accuracy in MCI [26]. We used a cut-off of < 68 points for the CERAD total score to define MCI. Paajanen et al. [27] used a CERAD total score of 100 points and found that the cut-off for MCI was < 69 points. In our study we used a version of CERAD in which the total points available in the figure drawing test were 10, with a CERAD total score of 99 points. Therefore, the cut-off for MCI was < 68 points. Among the 530 study participants, 56 (10.6%) had a CERAD total score < 68 and were defined as the MCI group.

A low cognitive performance at 57 years old was defined by results on at least two of the five tests being in the lowest 10% of the study population. Despite some missing data, there were enough test results from each of the five separate tests for each/subject to place the 530 participants into categories at baseline: lowest 10% in < 2 tests and lowest 10% in ≥ 2 tests. The cut-offs for the lowest 10% of the test results in the study population at baseline were ≤ 16 animals on the verbal fluency test (category), ≤11 words on the verbal fluency test (letter), ≤ 17 words on the word list learning test, ≥ 56 s for the TMA, and ≥ 142 s for the TMB. The respective cut-offs at follow-up were ≤ 15 animals, ≤ 6 words, ≤15 words, ≥ 66 s, and ≥ 200 s.

### Insulin resistance and glucose tolerance status

Baseline fasting glucose, fasting insulin, homeostasis model assessment of insulin resistance (HOMA-IR), and change in fasting insulin and HOMA-IR during the follow-up were measured. Insulin resistance was defined by the level of fasting insulin and HOMA-IR, which can be calculated from the fasting glucose and insulin levels by a mathematical model [28]: HOMA-IR = fasting glucose x fasting insulin/22.5.

The study population was divided into tertiles according to the change in HOMA-IR and fasting insulin level during the follow-up. The limits to the change (elevation) in HOMA-IR were ≥ 0.49 to < 1.50 mmol/L and to the change in insulin level were ≥ 2.00 to < 5.80 mU/L in the 2^nd^ tertile. The 1st tertile was below the lower boundary and the 3rd tertile was above the upper boundary of the 2nd tertile. An elevation of HOMA-IR and fasting insulin indicates progression of insulin resistance.

All participants without a previous diagnosis of diabetes mellitus were invited to participate in a 2-h oral glucose tolerance test (OGTT) after a 10-h fasting period, at both baseline and follow-up. Blood samples were obtained in the fasting state, immediately before the intake of 75 -g of glucose, and then 30, 60, and 120 min after the intake. The glucose tolerance status was classified according to WHO criteria for diabetes mellitus: diabetes = fasting plasma glucose level ≥ 7 mmol/L or 2-h plasma glucose level > 11 mmol/L; impaired fasting glucose (IFG) = fasting plasma glucose level 6.1–6.9 mmol/L; impaired glucose tolerance (IGT) = 2-h plasma glucose level 7.8–11 mmol/L; and NGT = fasting plasma glucose level ≤ 6.0 mmol/L and 2-h plasma glucose level < 7.8 mmol/L.

### Health related quality of life

We used a 15 D instrument to define the health-related quality of life in our study population [29]. We compared the differences in HRQoL scores between the non-MCI and MCI groups, as well as between individuals in different HOMA-IR tertiles (1st vs. combined 2nd and 3rd tertiles). When comparing the groups, we used an index score of 0.015 as a clinically significant difference [30].

### Ethical considerations and approvals

The follow-up study was approved by the Ethical Committee of the Northern Ostrobothnia Hospital District in Oulu, Finland (EETTMK 12/2013), and was performed in accordance with the Declaration of Helsinki. The participants were given oral and written information about the study, and all participants provided written consent to use data for research, including consent to link the data to hospital registers. The data were analyzed at a group level, and the personal details were replaced with identification codes.

### Statistical analysis

For comparisons between included (*n* = 530) and excluded (*n* = 184) participants, we used the chi‑squared test for categorical variables. We compared characteristics at baseline and follow-up using paired analysis. We used the McNemar test for dichotomous variables and McNemar-Bowker test for variables with more than two categories. For continuous variables, we used the paired samples t-test. Glucose status groups at baseline and follow-up were compared using the z-test for two proportions. Comparisons between the non-MCI and MCI-groups were achieved using the chi-squared test for categorical variables and the independent samples t-test or Mann-Whitney U-test for continuous variables, as appropriate. The linear association between insulin resistance and the CERAD scores was examined using scatterplots. We applied univariate and multivariate binary logistic regression to examine the association of changes in fasting insulin and HOMA-IR with MCI. In these analyses, the 1st tertiles of changes in fasting insulin and HOMA-IR were used as the reference categories and the 2nd and 3rd tertiles were combined as one category. As other explanatory variables in the final models, we chose cognitive performance at baseline, baseline education, and change in physical activity during follow-up. We based the selection of explanatory variables on our assessment of clinical relevance, as well as the significance of the variables in the univariate logistic regression analysis. Although sex did not reach statistical significance in the univariate analysis, we further assessed its potential influence by adding it to the multivariate models. In these adjusted models, sex remained non- significant, and the coefficients for the other explanatory variables were essentially unchanged. Estimates are presented as odds ratios (ORs) with 95% confidence intervals (CIs). Before multivariate model building the potential multicollinearity was assessed using the variance inflation factor (VIF). The Mann–Whitney U-test was used to compare the 15D dimensions of HRQoL between the non‑MCI and MCI groups, and likewise to compare the15D dimensions between the two HOMA‑IR groups, defined as the 1st tertile versus the combined 2nd and 3rd tertiles. Significance was set to *p* < 0.05. For clinically significant differences in 15D scores we deemed p-values between 0.05 and 0.07 as borderline significant. Statistical analyses were performed using SPSS 28 for Windows (Armonk, NY: IBM Corp).

## Results

The characteristics of the whole study population at baseline and follow-up are provided in Table 1. During the 12-year follow-up, self-reported lifestyle factors, such as smoking and alcohol consumption, decreased. In addition, the proportion of participants who performed physical activity less than once a week and the proportion of participants reporting clinically significant depressive symptoms decreased. However, the proportion of participants with hypertension (≥ 140/90, *p* < 0.001) and obesity (BMI ≥ 30 kg/m^2^, *p* < 0.001) increased. The incidence of dysglycemia increased during the follow-up, as 80.6% of the study population had NGT at baseline but only 42.3% had NGT at follow-up. The LDL cholesterol level did not change significantly during the follow-up. There was a significant decrease in all five cognitive tests used during the follow-up.

**Table 1.** The characteristics of the study population at baseline (age 57) and at follow-up (age 69).

Variable	Baseline (1)	Follow-up (1)	*p*-value (1)
**Sex**			
Male	209 (39.4%)		
Female	321 (60.6%)		
**Professional education**			
College/polytechnic/academy/university	221 (41.7%)		
Vocational school	115 (21.7%)		
No professional education/ courses	194 (36.6%)		
**Marital status *n* = 523**			0.005
Unmarried	72 (13.8%)	81 (15.5%)	
Married	367 (70.2%)	345 (60.0%)	
Widowed	22 (4.2%)	38 (7.3%)	
Divorced	62 (11.9%)	59 (11.3%)	
**Physical activity**			< 0.001
≤ 1 time/ wk	189 (35.7%)	121 (22.8%)	
> 1 time/ wk	341 (64.3%)	409 (77.2%)	
**Cognitive performance *n* = 528**			0.358
Lowest 10% in < 2 tests	462 (87.5%)	470 (89%)	
Lowest 10% in ≥ 2 tests	66 (12.5%)	58 (11%)	
**Cognitive test results**			
Verbal fluency test, category (words/min) n 529	25.7 (7.4)	22.3 (5.5)	< 0.001
Verbal fluency test, letter (words/ min) n 528	18.9 (5.9)	12.8 (5.2)	< 0.001
Word list learning (words) n 530	22.4 (3.7)	20.3 (3.8)	< 0.001
**Trail Making A (s) n 529**	38.6 (13.2)	45.2 (16.5)	< 0.001
**Trail Making B (s) n 508**	96.8 (36.5)	125.9 (62.9)	< 0.001
**Smoking status *n* = 508**			< 0.001
Nonsmoker	274 (53.9%)	292 (57.5%)	
Ex-smoker	145 (28.5%)	166 (32.7%)	
Current smoker	89 (17.5%)	50 (9.8%)	
**Alcohol consumption in doses /wk *n* = 513**			< 0.001
0	60 (11.7%)	96 (18.7%)	
≤14 in males, ≤ 7 in females	378 (73.7%)	364 (71%)	
>14 in males, > 7 in females	75 (14.6%)	53 (10.3%)	
Beck Depression Inventory *n* = 470			0.004
<10 points	368 (78.3%)	394 (83.8%)	
≥10 points	102 (21.7%)	76 (16.2%)	
**Body mass index (BMI)**			< 0.001
<25 kg/m (2)	196 (37%)	161 (30.4%)	
25–29.99 kg/m (2)	231 (43.6%)	236 (44.5%)	
≥30 kg/m (2)	103 (19.4%)	133 (25.1%)	
**Blood pressure**			< 0.001
<140 /90 mmHg	274 (51.7%)	181 (34.2%)	
≥140 /90 mmHg	256 (48.3%)	349 (65.8%)	
**LDL cholesterol**			< 0.001
<3 mmol/L	135 (25.5%)	183 (34.5%)	
≥3 mmol/L	395 (74.5%)	347 (65.5%)	
**Glucose status** ^3^			
NGT	427 (80.6%)	224 (42.3%)	< 0.001
IFG	31 (5.8%)	26 (4.9%)	0.496
IGT	59 (11.1%)	140 (26.4%)	< 0.001
IFG + IGT	13 (2.5%)	53 (10.0%)	< 0.001
Diabetes at follow-up (3)		87 (16.4%)	

^1^ Values are given as n (%) or mean (SD).

^2^ McNemar test for dichotomous variables and McNemar-Bowker test for variables with more than two categories. For glucose status, we used z-test for two proportions.

^3^ Based on OGTT or self-reported data when diabetes was present at follow-up. Diabetes at baseline excluded.

NGT: normal glucose tolerance; IFG: impaired fasting glucose; IGT: impaired glucose tolerance.

Tables 2–4 compares the characteristics of non-MCI and MCI groups, which were defined based on their CERAD total scores at follow-up. There was 56 individuals (29 female, 27 male) in the MCI group. Table 2. presents the sociodemographic and lifestyle characteristics, Table 3. presents the cognitive and mental characteristics, and Table 4. presents the metabolic and cardiovascular characteristics of the study population. A lower level of physical activity was significantly associated with MCI (*p* = 0.036). In addition, the level of cognitive performance at baseline was associated with MCI (*p* < 0.001). The change in fasting insulin and HOMA-IR was significant in both groups, and the fasting insulin level increased notably in both groups during the follow-up. The cognitive test results declined in both groups for all tests used. There was a significant decline in the word list learning test in the MCI group compared to the non-MCI group (*p* = 0.003).

**Table 2.** Baseline and follow-up sosiodemografic and lifestyle characteristics of the non-MCI and MCI groups.

	MCI at follow-up			*p*-value^2^	
Variable	Non-MCI (*n* = 474)	MCI (*n* = 56)	*p*-value^1^	Non-MCI	MCI
**Sex**			0.155	N/A	N/A
Male	182 (38.4 %)	27 (48.2 %)			
Female	292 (61.6 %)	29 (51.8 %)			
**Professional education**			<0.001	N/A	N/A
College/polytechnic/academy/univ. (>12 y)	212 (44.7 %)	9 (16.1 %)			
Vocational school (9–12 y)	107 (22.6 %)	8 (14.3 %)			
No professional education/courses (9 y)	155 (32.7 %)	39 (69.6 %)			
**Marital status (*n* = 523)**				0.003	0.416
**Baseline**			0.288		
Unmarried	60 (12.8%)	12 (21.4%)			
Married	330 (70.7%)	37 (66.1%)			
Widowed	21 (4.5%)	1 (1.8%)			
Divorced	56 (12.0%)	6 (10.7%)			
**Follow-up**			0.225		
Unmarried	69 (14.8%)	12 (21.4%)			
Married	307 (65.7%)	38 (67.9%)			
Widowed	37 (7.9%)	1 (1.8%)			
Divorced	54 (11.6%)	5 (8.9%)			
**Physical activity**				< 0.001	0.263
**Baseline**			0.138		
≤1 times/ week	164 (34.6%)	25 (44.6%)			
>1 times/ week	310 (65.4%)	31 (55.4%)			
**Follow-up**			0.036		
≤1 times/ week	102 (21.5%)	19 (33.9%)			
>1 times/ week	372 (78.5%)	37 (66.1%)			
**Smoking status**				< 0.001	0.029
**Baseline**			0.240		
Non-smoker	252 (55.1%)	22 (43.1%)			
Ex-smoker	128 (28.0%)	17 (33.3%)			
Current smoker	77 (16.8%)	12 (23.5%)			
**Follow-up**			0.609		
Non-smoker	266 (58.2%)	26 (51 %)			
Ex-smoker	147 (32.2%)	19 (37.3%)			
Current smoker	44 (9.6 %)	6 (11.8%)			
**Alcohol consumption in doses/wk (*n* = 513)**				< 0.001	0.059
**Baseline**			0.062		
0	53 (11.5%)	7 (13.2%)			
≤14 in males, ≤ 7 in females	334 (72.6%)	44 (83 %)			
>14 in males, > 7 in females	73 (15.9 %)	2 (3.8%)			
**Follow-up**			0.064		
0	81 (17.6 %)	15 (28.3%)			
≤14 men, ≤ 7 women	328 (71.3%)	36 (67.9%)			
Men > 14 / women > 7	51 (11.1 %)	2(3.8%)			

MCI was defined at follow-up according to the criteria stated in Methods section.

Values are given as n (%) or mean (SD) unless otherwise noted.

^1^ *p*-value for the difference between the groups. Chi-squared test for categorical variables and Student’s t-test or Mann-Whitney U-test for continuous variables as appropriate.

^2^ *p*-value for the change from baseline to follow-up. McNemar test or McNemar-Bowker test for categorical variables, as appropriate. Paired samples t-test for continuous variables. For glucose tolerance status, we used z-test for two proportions.

**Table 3.** Baseline and follow-up cognitive and mental characteristics of the non-MCI and MCI groups.

	MCI at follow-up			*p*-value (2)	
Variable	Non-MCI (*n* = 474)	MCI (*n* = 56)	*p*-value (1)	Non-MCI	MCI
**Cognitive performance**				0.108	0.648
**Baseline (*n* = 530)**					
Lowest 10% in < 2 tests	433 (91.4%)	30 (53.6%)	<0.001		
Lowest 10% in ≥ 2 tests	41 (8.6%)	26 (46.4%)	<0.001		
**Follow-up (*n* = 528)**					
Lowest 10% in < 2 tests	443 (93.7%)	27 (49.1%)	<0.001		
Lowest 10% in ≥ 2 tests	30 (6.3%)	28 (50.9%)	<0.001		
**Cognitive test results**					
**Baseline**					
Verbal fluency test, category (words/min)	26.5 (7.1)	19.3 (6.5)	< 0.001	< 0.001	< 0.001
Verbal fluency test, letter (words/min)	19.2 (5.8)	15.6 (5.7)	< 0.001	< 0.001	< 0.001
Word list learning (words)	22.8 (3.3)	18.5 (4.1)	< 0.001	< 0.001	< 0.001
Trail Making A (s)	37.7 (12.8)	45.5 (14.9)	< 0.001	0.001	< 0.001
Trail Making B (s)	94.3 (34.9)	119.7(42.7)	< 0.001	< 0.001	< 0.001
**Follow-up**					
Verbal fluency test, category (words/min)	23.1 (5.8)	15.5 (3.8)	< 0.001		
Verbal fluency test, letter (words/min)	13.2 (5.2)	9.2 (3.5)	< 0.001		
Word list learning (words/min)	20.9 (3.3)	14.6 (3.1)	< 0.001		
Trail Making A (s)	44.0 (15.3)	55.6 (21.7)	< 0.001		
Trail Making B (s)	119.9 (55.9)	169.9(91.6)	< 0.001		
**Beck Depression Inventory (*n* = 470)**				0.003	1.0
**Baseline**			0.771		
<10 points	332 (78.1%)	36 (80%)			
≥10 points	93 (21.9%)	9 (20%)			
**Follow-up**			0.463		
<10 points	358 (84.2%)	36 (80%)			
≥10 points	67 (15.8%)	9 (20%)			

MCI was defined at follow-up according to the criteria stated in Methods section.

Values are given as n (%) or mean (SD) unless otherwise noted..

^1^ *p*-value for the difference between the groups. Chi-squared test for categorical variables and Student’s t-test or Mann-Whitney U-test for continuous variables as appropriate.

^2^ *p*-value for the change from baseline to follow-up. McNemar test or McNemar-Bowker test for categorical variables, as appropriate. Paired samples t-test for continuous variables. For glucose tolerance status, we used z-test for two proportions.

**Table 4.** Baseline and follow-up metabolic and cardiovascular characteristics of the non-MCI and MCI groups.

	MCI at follow-up			*p*-value (2)	
Variable	Non-MCI (*n* = 474)	MCI (*n* = 56)	*p*-value (1)	Non-MCI	MCI
**Body mass index (BMI)**				< 0.001	0.375
**baseline**			0.444		
< 25 kg/m (2)	171 (36.1%)	25 (44.6%)			
25-29.99 kg/m (2)	209 (44.1%)	22 (39.3%)			
≥ 30 kg/m (2)	94 (19.8%)	9 (16.1%)			
**follow-up**			0.415		
< 25 kg/m (2)	142 (30%)	19 (33.9%)			
25-29.99 kg/m (2)	209 (44.1%)	27 (48.2%)			
≥ 30 kg/m (2)	123 (25.9%)	10 (17.9%)			
**Blood pressure**				< 0.001	0.210
**Baseline**			0.404		
<140/90 mmHg	248 (52.3%)	26 (46.4%)			
≥140/90 mmHg	226 (47.7%)	30 (53.6%)			
**Follow-up**			0.970		
<140/90 mmHg	162 (34.2%)	19 (33.9%)			
≥140/90 mmHg	312 (65.8%)	37 (66.1%)			
**LDL cholesterol**				0.001	0.267
**Baseline**			0.463		
<3.0 mmol/L	123 (25.9%)	12 (21.4%)			
≥3.0 mmol/L	351 (74.1%)	44 (78.6%)			
**Follow-up**			0.488		
<3.0 mmol/L	166 (35%)	17(30.4%)			
≥3.0 mmol/L	308 (65%)	39(69.6%)			
**Fasting glucose**, mmol/L				< 0.001	0.137
Baseline	5.35 (0.54)	5.37 (0.57)	0.800		
Follow-up	5.63 (0.84)	5.50 (0.65)	0.184		
**fFasting insulin**, mU/l, median (IQR)				< 0.001	< 0.001
Baseline	5.60 (7.90, 11.0)	5.30 (6.90, 9.53)	0.087		
Follow-up	11.70 (8.30, 16.13)	12.65 (9.65,17.73)	0.203		
**HOMA-IR,** mmol/L, median (IQR)				< 0.001	<0.001
Baseline	1.87 (1.32, 2.69)	1.63 (1.24, 2.31)	0.149		
Follow-up	2.81 (1.95, 4.25)	3.17 (2.20, 4.29)	0.337		
**Change in fasting glucose during the follow-up**			0.674	N/A	N/A
1st tertile	156 (32.9%)	20 (35.7%)			
Combined 2nd and 3rd tertile	318 (67.1%)	36 (64.3%)			
**Change in fasting insulin during the follow-up**			<0.001	N/A	N/A
1^st^ tertile	168 (35.4%)	7 (12.5%)			
Combined 2^nd^ and 3^rd^ tertile	306 (64.6%)	49 (87.5%)			
**Change in HOMA-IR during the follow-up**			0.023	N/A	N/A
1^st^ tertile	165 (34.8%)	11 (19.6%)			
Combined 2^nd^ and 3^rd^ tertile	309 (65.2%)	45 (80.4%)			
**Glucose tolerance status**				N/A	N/A
**Baseline**			0.501		
NGT	380 (80.2%)	47 (83.9%)			
Prediabetes (IFG, IGT, IFG + IGT)	94 (19.8%)	9 (16.1%)			
**Follow-up**			0.237		
NGT	204 (43%)	20 (35.7%)			
Prediabetes (IFG, IGT, IFG + IGT)	190 (40.1%)	29 (51.8%)			
Diabetes at follow-up	80 (16.9%)	7 (12.5%)			

MCI was defined at follow-up according to the criteria stated in Methods section.

Values are given as n (%) or mean (SD) unless otherwise noted.

^1^ *p*-value for the difference between the groups. Chi-squared test for categorical variables and Student’s t-test or Mann-Whitney U-test for continuous variables as appropriate.

^2^ *p*-value for the change from baseline to follow-up. McNemar test or McNemar-Bowker test for categorical variables, as appropriate. Paired samples t-test for continuous variables. For glucose tolerance status, we used z-test for two proportions.

HOMA-IR: Homeostasis Model Assessment of Insulin Resistance; NGT: normal glucose tolerance; IFG: impaired fasting glucose; IGT: impaired glucose tolerance.

**Figure 2.**
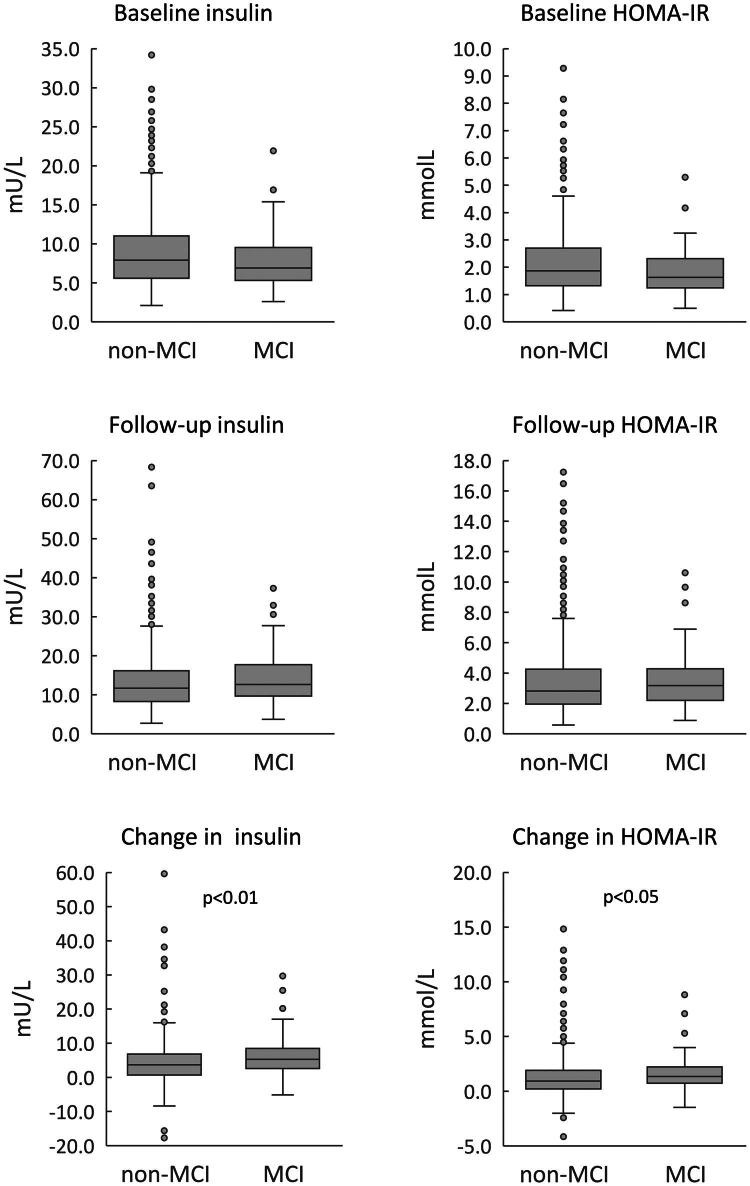
Boxplots of insulin and HOMA-IR levels across the non-MCI and MCI groups. One participant in the non‑MCI group and one participant in the MCI group had an extreme outlier value for follow‑up insulin. As a result, two values fall outside the visible plotting area in the figures depicting follow‑up insulin, follow‑up HOMA‑IR, and the corresponding changes.

The baseline fasting insulin level or HOMA-IR was not associated cross-sectionally with MCI at follow-up. However, during the follow-up 87.5% of the participants in the MCI group settled in higher tertiles of the change in fasting insulin, which is a significant difference from the non-MCI group (64.6%). Similarly, 80.4% of the participants in the MCI group settled into higher tertiles of the change in HOMA-IR, which was a significant difference from the non-MCI group (65.2%). No linear association was observed between insulin resistance and the CERAD results in the scatterplots. The boxplot figures for insulin and HOMA-IR across the non-MCI and MCI groups are shown in Figure 2.

The glucose tolerance status was not associated with MCI at follow-up.

To further examine the association between insulin resistance and the risk of MCI, we conducted univariate and multivariate logistic regression analyses (Table 5). Belonging to higher tertiles of the increase in insulin resistance and fasting insulin during follow-up increased the risk of MCI at 69 years of age 2-fold (OR 2.20, 95% CI 1.05– 4.62) and 3.5-fold (OR 3.49, 95% CI 1.48–8.23, respectively). A lower level of cognitive performance at baseline (OR 6.33, 95% CI 3.27–12.29), lower level of education (OR 3.94, 95% CI 1.76–8.81), and lower level of physical activity during follow-up (OR 2.44, 95% CI 1.06–5.65) adjusted for the change in HOMA-IR independently increased the risk of MCI at follow-up. Adjustment for the change in fasting insulin gave similar results. The constant variables were level of education, physical activity and multiple laboratory parameters in models I to III. Including sex in the models of adjustment did not change the result.

**Table 5.** Odds ratios for the risk of MCI according to potential explanatory variables.

Variable	Crude OR (95% CI)	Model I OR (95 % CI)	Model II OR (95% CI)	Model III OR (95% CI)
**Professional education**				
College/polytechnic/academy/ university	1.0 (reference)	1.0 (ref)	1.0 (ref)	1.0 (ref)
No education/courses	5.93 (2.79–12.60)	3.83 (1.72–8.54)	3.93 (1.75–8.84)	3.94 (1.76–8.81)
Vocational school	1.76 (0.66–4.69)	1.40 (0.51–3.88)	1.37 (0.49–3.81)	1.39 (0.50–3.86)
**Physical activity during follow-up**				
Remained > 1 time / week	1.0 (reference)	1.0 (ref)	1.0 (ref)	1.0 (ref)
Remained ≤ 1 time /week	2.23 (1.06–4.72)	2.40 (1.04–5.50)	2.52 (1.07–5.90)	2.44 (1.06–5.65)
Decreased to ≤ 1 time/ week	1.86 (0.76–4.59)	1.57 (0.58–4.23)	1.60 (0.59–4.35)	1.51 (0.55–4.12)
Increased > 1 time / week	1.40 (0.69–2.85)	1.54 (0.71–3.36)	1.46 (0.66–3.23)	1.48 (0.68–3.24)
**Cognitive performance at baseline**				
Lowest 10 % in < 2 tests	1.0 (reference)			
Lowest 10 % in ≥ 2 tests	9.15 (4.95–16.93)	6.45 (3.34–12.46)	5.89 (3.02–11.49)	6.33 (3.27–12.29)
**Change in fasting insulin**				
1^st^ tertile	1.0 (reference)			
Combined 2^nd^ and 3^rd^ tertile	3.84 (1.70–8.67)		3.49 (1.48–8.23)	
**Change in HOMA-IR**				
1^st^ tertile	1.0 (reference)			
Combined 2^nd^ and 3^rd^ tertile	2.18 (1.10–4.34)			2.20 (1.05–4.62)

The cut-off values for poor cognitive test results were the lowest 10% for the study population at baseline (age 57 years).

Model I includes cognitive performance at baseline, professional education, and physical activity.

Model II includes cognitive performance at baseline, professional education, physical activity and change in fasting insulin during the follow-up.

Model III includes cognitive performance at baseline, professional education, physical activity and change in HOMA-IR during the follow-up.

HOMA-IR: Homeostasis Model Assessment of Insulin Resistance.

**Figure 3.**
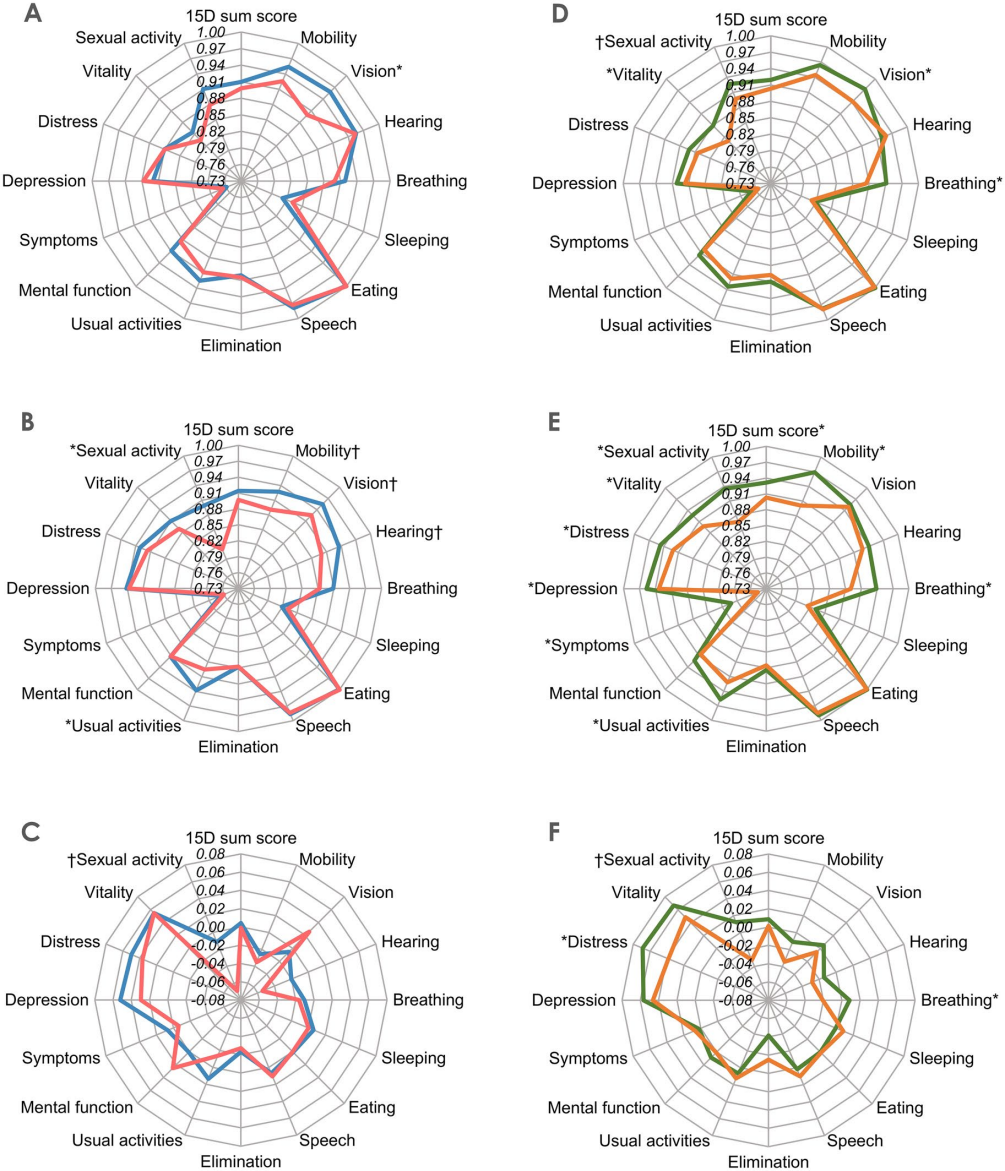
15D dimensions at 57 years of age (panel A) and 69 years of age (panel B), and the change in D15 dimensions during follow-up (panel C). The blue line indicates the non-MCI group, and the red line indicates the MCI group. Panels D, E, and F illustrate the effect of insulin resistance on 15D dimensions. The green line represents the lowest tertile of HOMA-IR, and the orange line represents the 2nd and 3rd tertiles combined. Panel D presents baseline HOMA-IR, Panel E follow-up HOMA-IR, and Panel F the change in HOMA-IR. *p < 0.05, †*p* < 0.07.

Figure 3 illustrates HRQoL outcomes, where larger circles indicate better status. At baseline (panel A), differences between the non-MCI and MCI groups were minimal, apart from a significant difference in Vision (*p* < 0.05). By follow-up (panel B), group differences had increased, with Usual Activities and Sexual Activity significantly poorer in the MCI group (*p* < 0.05), and borderline differences in Mobility, Vision, and Hearing (*p* < 0.07). Panel C shows that HRQoL declined more across most dimensions in the MCI group, with a borderline significant decline in Sexual Activity (*p* < 0.07).

Panels D, E, and F illustrate the effect of insulin resistance on 15D dimensions of HRQoL the green line representing the lowest tertile of HOMA-IR, and the orange line representing the 2nd and 3rd tertiles combined. The baseline HOMA-IR is presented in panel D and the follow-up HOMA-IR in panel E. The effect of the change in HOMA-IR on 15D dimensions is presented in panel F. At baseline, individuals in the higher HOMA-IR tertiles had significantly poorer scores (*p* < 0.05) in Breathing, Vision, and Vitality. At follow-up, they had significantly poorer scores (*p* < 0.05) in Breathing, Depression, Distress, Mobility, Sexual activity, Symptoms, Usual activities, and Vitality. The 15D total score at follow-up was also significantly poorer (*p* < 0.05) among those in the higher HOMA-IR tertiles. The decline in 15D scores over the follow-up period was significant (*p* < 0.05) in Breathing and Distress, and borderline significant (*p* < 0.07) in Sexual activity.

## Discussion

In this longitudinal study spanning 12 years, we investigated the changes in cognitive performance and risk factors for MCI in a non-diabetic cohort at baseline, with a focus on individuals who were 69 years old. The prevalence of MCI, determined by a CERAD score cut-off of 68 points, exceeded 10%, which aligns with prevalences reported in prior studies utilizing various methodologies.

As exacerbation of insulin resistance or increase in fasting plasma insulin levels heightened the risk of MCI, even after adjusting for educational background, cognitive test outcomes, and physical activity levels. Interestingly, none of the potential cardiovascular risk factors exhibited an independent association with MCI after 12 years. However, low education levels and impaired performance on cognitive tests at baseline significantly correlated with subsequent MCI.

The relationship between cognitive impairment, including dementia, and diabetes (both type 1 and type 2) is well-documented, with similar associations reported for prediabetes [1–3]. Our research noted a marked increase in the incidence of dysglycemia throughout the follow-up period. Clinically, this suggests that the deterioration of glucose metabolism with aging warrants consideration due to its apparent ubiquity. The risk of MCI has not been examined extensively. Notably, individuals with MCI face a heightened risk of developing dementia, though this likelihood varies based on the definition of MCI employed across studies[31].

Previous cross-sectional research explored the link between insulin resistance and cognitive decline [7–12]. Most studies have focused on the association of insulin resistance with diagnosed Alzheimer’s disease or dementia, but also the connection to cognitive impairment has been assessed longitudinally. Tortelli et al. [17] defined MCI and categorized their study participants into three groups based on MMSE scores: normal cognition, mild cognitive decline, and moderate/severe cognitive decline. They observed a significant increase in the HOMA-IR when comparing the normal cognition group to the moderate/severe cognitive decline group but found no correlation between MCI and insulin resistance. Other studies did not define MCI. Young et al. [13] had a 6-year follow-up and found that higher fasting insulin and HOMA-IR at baseline were associated with greater decline in delayed word recall and word fluency tests. Ekblad et al. [14] found that higher baseline HOMA-IR predicted a greater decline in verbal fluency. Kong et al. [15] used the MMSE and found, during 6-year follow-up, that increased insulin resistance was associated with poorer cognitive performance. Hooshmand et al. [16] reported that, after excluding cases of incident dementia, higher baseline HOMA-IR and elevated serum insulin levels were associated with poorer global cognitive performance after 7 years.

The etiology of cognitive decline is multifactorial, with various environmental conditions potentially influencing molecular mechanisms. Our findings indicate that lower education, suboptimal cognitive test performance in middle age, and reduced physical activity levels increase MCI risk. The Finnish professional education system, which differed historically from today’s structure, categorized ‘no education’ as the absence of professional education, ‘vocational school’ as career college, and the highest professional education group included secondary school degrees, previously classified as career college degrees. Within our study cohort, individuals with lower levels of professional education exhibited reduced cognitive abilities at 57 years old, a period when pathological cognitive dysfunction is uncommon. This cohort, which was born in 1945, generally had lower education levels due to the socioeconomic conditions of the time.

A sedentary lifestyle is posited to be a risk factor for cognitive decline [32] and starting regular physical activity even if later in life seems to benefit in lowering the MCI risk [33]. In our study we categorized the participants into two groups, those without physical activity and those with at least minimal physical activity, to assess whether even modest activity could serve as a protective factor against MCI. We found this minimal physical activity to be an independent risk factor for later MCI.

The relationship between insulin resistance and the neuropathology of cognitive function, as well as Alzheimer’s disease progression, is complex. Insulin supports brain health by regulating energy metabolism, enhancing synaptic plasticity, promoting dendritic spine formation, and regulating neurotransmitter turnover. Through these mechanisms, insulin influences amyloid-β clearance, tau phosphorylation, vasoreactivity, lipid metabolism, and neuroinflammation. Consequently, insulin dysregulation has increasingly been linked to neurodegenerative processes [11]. Prior research identified links between cerebrospinal fluid (CSF) biomarkers, specifically amyloid β, total tau (t-tau), and phosphorylated tau (p-tau), and the advancement of Alzheimer’s pathology. Woodfield et al. [34] demonstrated that reduced CSF Aβ42 levels, in conjunction with elevated HOMA-IR, are associated with increased CSF t-tau and p-tau concentrations. Insulin resistance is known to correlate with lower levels of serum adiponectin, an integral protein to glucose regulation [35]. Cross-sectional analyses indicated that HOMA-IR is associated with MCI or cognitive decline in both diabetic and non-diabetic individuals [36–40]. Some studies have suggested that this association may be linked to the apolipoprotein E genotype and vascular mechanisms.

The association of MCI and health related quality of life has not been investigated thoroughly but we observed that several indicators in the 15D analysis had weaker scores or greater decline among those with later MCI. In addition, individuals with higher HOMA-IR values (indicating greater insulin resistance) had poorer 15D test scores. Those in the highest HOMA-IR tertiles already showed poorer 15D scores at baseline, and the differences were even more pronounced at the follow-up assessment. It can be discussed whether MCI affects HRQoL due to an increased need for assistance, and whether the poorer HRQoL associated with insulin resistance may be a consequence of comorbid conditions related to insulin resistance or their symptoms.

The present study’s strengths include its longitudinal design and extensive 12-year follow-up period. In addition, the CERAD test, a clinically relevant cognitive assessment, was employed to define MCI. Furthermore, insulin resistance was measured using both HOMA-IR and fasting insulin levels, and the study also examined the impact of increasing insulin resistance over the duration of the follow-up.

A potential limitation of this study is the population-based design, which is susceptible to selection bias during follow-up [41]. Individuals with poorer health and well-being may be under-represented at baseline: thus, the results may not be generalizable to comorbid populations. Of the 714 participants, data on CERAD results and insulin resistance measurements were available for 530, meaning that 26% of the cohort was absent from our analysis concerning the association between insulin resistance and cognitive performance, which could influence the findings. Among those who were excluded due to missing data, there were slightly more individuals without professional education and fewer with normal glucose status compared with the study population. It is possible that the findings regarding cognitive decline might have been even more pronounced had these individuals been included in the analyses.

In conclusion, this study posits that increased insulin resistance accelerates cognitive decline with aging. In addition, the results suggest that avoidance of a sedentary lifestyle among the elderly could protect against cognitive decline. These insights should be considered in clinical settings as further justification for promoting a healthy lifestyle to prevent insulin resistance. General practitioners play a key role in identifying individuals at risk of insulin resistance and motivating them to make the necessary lifestyle changes to prevent associated diseases.
